# Are integrated HIV services less stigmatizing than stand-alone models of care? A comparative case study from Swaziland

**DOI:** 10.7448/IAS.16.1.17981

**Published:** 2013-01-11

**Authors:** Kathryn Church, Alison Wringe, Phelele Fakudze, Joshua Kikuvi, Dudu Simelane, Susannah H Mayhew, The Integra Initiative

**Affiliations:** 1 Department of Global Health and Development, London School of Hygiene and Tropical Medicine, London, UK; 2 Population Studies Department, London School of Hygiene and Tropical Medicine, London, UK; 3 Family Life Association of Swaziland, Manzini, Swaziland; 4 London School of Hygiene and Tropical Medicine, c/o Family Life Association of Swaziland, Manzini, Swaziland

**Keywords:** systems integration, health services research, stigma, HIV, primary care, reproductive health services

## Abstract

**Introduction:**

Integrating HIV with primary health services has the potential to reduce HIV-related stigma through delivering care in settings disassociated with HIV. This study investigated the relationship between integrated care and felt stigma. The study design was a comparative case study of four models of HIV care in Swaziland, ranging from fully integrated to fully stand-alone HIV care.

**Methods:**

An exit survey (*N*=602) measured differences in felt stigma across model of care; the primary outcome “perception of HIV status exposure through clinic attendance” was analyzed using multivariable logistic regression. In-depth interviews (*N*=22) explored whether and how measured differences in stigma experiences were related to service integration.

**Results:**

There were significant differences in perceived status exposure across models of care. After adjustment for potential confounding between sites, those at a partially integrated site and a partially stand-alone site had greater odds of perceived status exposure than those at the fully stand-alone site (aOR 3.33, 95% CI 1.98–5.60; and aOR 11.84, 95% CI 6.89–20.36, respectively). There was no difference between the fully stand-alone and the fully integrated clinic. Qualitative data suggested that many clients at HIV-only sites felt greater confidentiality knowing that those around them were positive, and support was gained from other HIV care clients. Confidentiality was maintained in various ways, even in stand-alone sites, through separate waiting areas for HIV testing and HIV treatment, and careful clinic and room labelling.

**Conclusions:**

The relationship between model of care and stigma was complex, and the hypothesis that stigma is higher at stand-alone sites did not hold true in this high prevalence setting. Policy-makers should ensure that service integration does not increase stigma, in particular within partially integrated models of care.

## Introduction

Integrating HIV services with generalist primary health care (PHC), including sexual and reproductive health (SRH) care, has the potential to reduce HIV-related stigma experienced within healthcare settings [1–5]. While health services can act as a locus of stigma in various ways [6], including through discriminating behaviour by health professionals, the structural organization of care may play a role through the protection of client HIV status and confidentiality.

Defined as a process of devaluation of people either living with HIV (PLWH), or associated with the illness, stigma is often followed by discrimination, the unjust treatment of individuals based on their real or perceived HIV status [7]. It is often conceptualized as “felt,” stemming from PLWH's negative perceptions about themselves, or “enacted,” involving the discriminatory behaviour of others [8]. Reduction of stigma is a critical goal for HIV programming due to its influence on health service utilization and drug adherence [9–12].

Most research on stigma in health settings focuses on the role of health workers as perpetrators of stigma. A recent review identified various discriminatory behaviours, including differential treatment, denial of care, testing or status disclosure without consent, verbal abuse, gossip, additional fees, and overuse of gloves [6]. Research on the influence of structural factors, including the effect of service integration or specialization, is more limited [13]. It has been speculated that stand-alone HIV services may be particularly stigmatizing, as clients are labelled as they walk through the door [14], resulting in an involuntary disclosure of status. Other structural influences include avoidance or isolation of HIV clients, and labelling of buildings and rooms [6, 14].

Evidence on the impact of service integration on stigma within PHC and SRH settings is equivocal. Descriptive studies suggest that integrated services may offer a less stigmatizing environment due to perceived anonymity [15–18], in contrast to a stressful “othering” process that has been identified in specialized settings [19, 20]. However, confidentiality breeches have been reported when HIV clients were mixed with SRH clients in waiting rooms [21], and some types of clients may desire specialization to enhance privacy: a comparison of vertical and integrated sexually transmitted infection services found higher utilization rates in specialist sites, partly reflecting these needs [22]. Some PLWH may also prefer specialized HIV care due to enacted provider stigma within SRH services [23, 24]. Furthermore, descriptive studies following service integration report that privacy is not always maintained in generalist settings, calling into question the supposed confidentiality of an integrated approach [17, 25, 26].

Since both felt and enacted stigma remain pervasive in high HIV prevalence contexts in sub-Saharan Africa, scaling-up models of care that minimize service-related stigma is critical. In Swaziland, the country with the world's highest HIV prevalence (26% among adults) [27], studies document stigmatizing attitudes and behaviours towards PLWH in the community, workplace, family, and by sexual partners [8, 28–31]. Policy-makers there have anticipated that by integrating HIV with primary care, this will lead to reductions in HIV-related stigma [32, 33]. This study aimed to explore experiences of HIV stigmatization across four different models of HIV care, and was part of a broader comparative case study investigating the process and outcomes of integrating SRH and HIV services in Swaziland [34]. It also formed a sub-component of a multi-country study investigating the benefits and costs of service integration, the “Integra Initiative,” [35] ClinicalTrials.gov identifier: NCT01694862.

## Methods

### Design and setting

A comparative case study design, using mixed quantitative and qualitative methods, investigated service provision and client experiences in real-life settings [36, 37]. Four case study clinics were identified within Swaziland's largest town, Manzini, to represent specific models of integrated or stand-alone HIV service delivery accessible to the same catchment population (all provided anti-retroviral therapy (ART) free of charge). The sites were the only HIV care facilities operational in Manzini at the time of the study and represented a continuum of service integration (see Table 1). Clinic A offered ART in the same consultation room as other SRH services (fully integrated); Clinic B offered ART in the same building as other SRH services (partially integrated); Clinic C offered ART in an outpatient clinic located on a district hospital campus (partially stand-alone); and Clinic D offered only ART and HIV testing services (fully stand-alone). Qualitative methods were used to help interpret the results of the quantitative component. The primary hypothesis was that felt stigma, as measured by perceived HIV status exposure through clinic attendance, would be lowest within the fully integrated model of care and highest at the fully stand-alone model of care.

**Table 1:** The four study clinics and their characteristics

	A: Fully integrated	B: Partially integrated	C: Partially stand-alone	D: Fully stand-alone
Integration/specialization	All SRH and HIV services offered by 1 provider in 1 consultation room.	Different providers offer SRH and HIV services in different consultation rooms in 1 building.	Providers offer HIV care services in a separate out-patient building on campus of district hospital; with referral to other hospital departments.	HIV only clinic offering care and testing services.
Type of clinic	NGO-run SRH clinic (primary/secondary care).	Government-run public health unit (PHU) (primary/secondary care).	Half government-/half mission-run district hospital (secondary/tertiary care).	NGO-run HIV clinic (primary/secondary care).
Services available in addition to HIV care*	FP, STI, ANC, PMTCT, PNC, VCT, pap smears, youth counselling.	FP, STI, ANC, PMTCT, PNC, VCT, pap smears, dental, skin.	Other HIV services only (VCT); though full range of in- and out-patient services available in other hospital buildings.	Other HIV services only (VCT).

* FP=family planning; STI=sexually transmitted infection services; ANC=antenatal care; MCH=maternal & child health; PMTCT=prevention of mother to child transmission services; PNC=postnatal care; VCT=voluntary counselling and testing of HIV.

### Quantitative methods

An exit survey was conducted in 2009 among male and female HIV care clients (*N*=602) (pre-ART or on ART) aged 18 and above. A structured questionnaire administered by trained fieldworkers asked respondents about comfort in the clinic as a PLWH, perceived HIV status exposure risk and preferences for stand-alone HIV care, as well as socio-demographic and health-related indicators. A conceptual model was developed based on the existing literature, formative interviews with clients and providers, and in-depth knowledge of the research context. Potential confounders that may influence perceptions of stigma included socio-economic variables (sex, age, marital status, education, income, religion, employment status), geographic (travel cost to clinic), SRH factors (number of sex partners in past month, number of living children, current pregnancy), health factors (type of client, time enrolled at clinic, whether or not on ARV, CD4 count and TB treatment status), and psycho-social factors (client comfort in waiting room and abandonment by partner due to HIV status). Questionnaires were administered in SiSwati following each client's completion of service contacts at the clinic. Systematic random sampling was used to identify clients as they exited the facilities, with intervals based on client load and interviewer capacity. At integrated sites, a ticketing system co-administered by nurses or receptionists was used to discreetly invite HIV clients for interview.

The primary outcome, fear of HIV status exposure through clinic attendance, was measured using a Likert scale, with respondents asked to rate agreement with the statement “Others can find out my status when I come here,” as follows: 1 “strongly disagree,” 2 “disagree,” 3 “mixed feelings, 4 “agree” and 5 “strongly agree.” The outcome was categorized into a binary variable for hypothesis testing (mean fear of status exposure score≥4 out of 5) due to substantial end skew in the scaled data.

STATA 10.0 was used for data checking, cleaning and analysis. Since personal digital assistants were used for data collection, there was only one instance where an explanatory variable contained missing data, and the population median was assigned to the case. A crude analysis examined the association between clinic model and perception of status exposure through clinic attendance, as well as between potential confounders and both exposure and outcome, using the χ^2^ test for categorical variables or analysis of variance (ANOVA) for continuous variables. Stratum-specific odds ratios of the association between clinic model and outcome were tabulated across potential confounders in a bivariate analysis, using the Mantel-Haenszel method: no significant interaction was identified. Logistic regression modelling was used to analyze the association between clinic model and perceived status exposure. A parsimonious regression model was identified via backward-fitting regression techniques and confirmed using forward-fitting regression techniques (using the likelihood ratio test).

A further sensitivity analysis was conducted to test for residual confounding in baseline data around perceptions of stigmatization that were independent of a clinic model, since only two such variables were evaluated as confounders. A second model, limited to those with a regular partner (*N*=490), included the variable “disclosed to partner.” However, as this did not improve the fit of the data to the model, the whole sample was used.

Ethical approval was obtained from the Ethical Committee at the London School of Hygiene & Tropical Medicine (approval no. 5436) and from the Swaziland Scientific and Ethics Committee (approval no. MH/139).

### Qualitative methods

In-depth interviews (IDIs) were conducted with 22 HIV clients (5–7 per clinic), interviewed three times on the day of ART initiation, and then two and six months later. Six clients were lost to follow-up at rounds 2 and 3. Clients were sampled both purposively and opportunistically: respondents were invited for interview during adherence counselling by the research team or by counsellors. The aim was to interview at least five per clinic, but potentially more to achieve data saturation. Those exiting first and/or when interviewers were available were interviewed, though efforts were made to include men and pregnant women. Interviews were conducted in SiSwati in private at clinics and follow-up at a local non-government organization or the participant's home. The interview topic guide covered client experiences in the clinic since initiating ART, their treatment by and interaction with providers (including any experiences of stigmatizing behaviour towards PLWH), access to and use of SRH services and attitudes towards integrated or stand-alone models of care. Interviews were recorded, transcribed and translated into English. Data were analyzed in the following iterative process: (i) data familiarization, through transcript review; (ii) coding framework development (facilitated by NVivo 8.0), derived deductively from the research questions and inductively from the data; (iii) abstraction of coded data into thematic matrices, allowing for a constant comparative approach across clinics and cases [38]; and (iv) interpretation, methodological synthesis (between qualitative and quantitative results.

## Results

### Quantitative results

Table 2 presents key characteristics of the survey sample, by clinic, composed of 127 male and 475 female clients. The refusal rate was 15.3%, which varied by clinic, being lowest at Clinics C and D (6% and 5%, respectively) and highest at A and B (22% and 28%, respectively). Client populations at the four sites differed significantly across the socio-demographic and health-related characteristics presented in Table 2. Notably, the largest group of clients were in their 30s (37%); the great majority were on ARVs (82%) and 39% reported a CD4 <200 cells/µl.

**Table 2:** Socio-demographic and health-related characteristics of exit survey sample (*N*=602)

	Clinic A	Clinic B	Clinic C	Clinic D	All clinics	
	
Variable	% (N)	% (N)	% (N)	% (N)	% (N)	P value (χ^2^)
Sex (female)	76.1 (54)	87.7 (143)	74.7 (133)	76.3 (145)	78.9 (475)	0.014
Age						
<25	22.5 (16)	28.8 (47)	10.7 (19)	8.9 (17)	16.4 (99)	<0.001
25–29	18.3 (13)	28.8 (47)	21.3 (38)	20.5 (39)	22.8 (137)	
30–39	42.3 (30)	33.1 (54)	38.2 (68)	38.4 (73)	37.4 (225)	
≥40	16.9 (12)	9.2 (15)	29.8 (53)	32.1 (61)	23.4 (141)	
Marital status (married)	47.9 (34)	58.9 (96)	47.2 (84)	50.5 (96)	51.5 (310)	0.149
Education						
No education	5.6 (4)	7.4 (12)	11.2 (20)	5.3 (10)	7.6 (46)	<0.001
0–7 years (primary)	14.1 (10)	30.7 (50)	35.4 (63)	18.9 (36)	26.4 (159)	
8–12 years (secondary)	54.9 (39)	59.5 (97)	49.4 (88)	69.5 (132)	59.1 (356)	
≥12 years (college)	25.4 (18)	2.5 (4)	3.9 (7)	6.3 (12)	6.8 (41)	
Household monthly income*						
E<500	19.7 (14)	28.8 (47)	49.4 (88)	28.9 (55)	33.9 (204)	<0.001
E500–999	18.3 (13)	32.5 (53)	27.5 (49)	21.1 (40)	25.7 (155)	
E1000–4999	35.2 (25)	37.4 (61)	21.3 (38)	36.8 (70)	32.2 (194)	
≥E5000	26.8 (19)	1.2 (2)	1.7 (3)	13.2 (25)	8.1 (49)	
Geographic location (cost from clinic)*						
E0–E5	32.4 (23)	60.7 (99)	37.6 (67)	44.7 (85)	45.5 (274)	<0.001
E6–E10	25.4 (18)	23.9 (39)	22.5 (40)	24.2 (46)	23.8 (143)	
E11–E20	21.1 (15)	9.2 (15)	30.9 (55)	19.5 (37)	20.3 (122)	
>E20	21.1 (15)	6.1 (10)	9.0 (16)	11.6 (22)	10.5 (63)	
Currently pregnant	19.7 (14)	32.5 (53)	7.3 (13)	4.7 (9)	14.8 (89)	<0.001
Client type						
Pre-ART	23.9 (17)	13.5 (22)	6.7 (12)	8.9 (17)	11.3 (68)	<0.001
ART initiation	11.3 (8)	5.5 (9)	4.5 (8)	0.0 (0)	4.2 (25)	
ART refill	50.7 (36)	49.1 (80)	73.6 (131)	77.9 (148)	65.6 (395)	
ART user consult	14.1 (10)	8.0 (13)	15.2 (27)	13.2 (25)	12.5 (75)	
PMTCT	0.0 (0)	23.9 (39)	0.0 (0)	0.0 (0)	6.5 (39)	
Time (months) enrolled at clinic						
<6 months	57.7 (41)	46.6 (76)	35.4 (63)	25.3 (48)	37.9 (228)	<0.001
6 months–2 years	26.8 (19)	42.3 (69)	28.1 (50)	66.3 (126)	43.9 (264)	
>2 years	15.5 (11)	11.0 (18)	36.5 (65)	8.4 (16)	18.3 (110)	
On ARVs	69.0 (49)	68.7 (112)	91.0 (162)	90.5 (172)	82.2 (495)	<0.001
Most recent CD4 count (cells/µl)						
<50	9.9 (7)	1.2 (2)	8.4 (15)	9.5 (18)	7.0 (42)	0.032
51–200	33.8 (24)	30.1 (49)	29.8 (53)	34.7 (66)	31.9 (192)	
>200	52.1 (37)	63.8 (104)	57.3 (102)	54.7 (104)	57.6 (347)	
No count	4.2 (3)	4.9 (8)	4.5 (8)	1.1 (2)	3.5 (21)	
On TB treatment	7.0 (5)	1.2 (2)	7.9 (14)	3.7 (7)	4.7 (28)	0.021
**Total**	**100.0 (71)**	**100.0 (163)**	**100.0 (178)**	**100.0 (190)**	**100.0 (602)**	

* Current in Swazi Emalangeni.

E11=£1.00 at the time of survey.

Table 3 presents measures of a priori “felt stigma” among respondents, which may also confound the association between clinic model and fear of status exposure. Most clients were not concerned if others in the waiting room knew their status (mean score=2.21 (out of 5.00), SD 1.5); however, those at the two integrated sites were more concerned: the proportion scoring ≥4 out of 5 ranged from 36.6% at the most integrated site (Clinic A) to 7.4% at the fully stand-alone site (Clinic D). Clients infrequently reported partner abandonment due to HIV (8%), and, among those with a regular partner (*N*=497), 13.3% had not disclosed their status; both of these did not vary significantly by clinic.

**Table 3:** Felt stigma among clients

	Clinic A	Clinic B	Clinic C	Clinic D	All clinics	P value
	
Variable	% (N)	% (N)	% (N)	% (N)	% (N)	(χ^2^)
Very bothered by others knowing status (score≥4/5)	36.6 (26)	32.5 (53)	19.1 (34)	7.4 (14)	21.1 (127)	<0.001
Abandoned by a partner due to HIV status	5.6 (4)	4.9 (8)	11.2 (20)	8.9 (17)	8.1 (49)	0.150
**Total (all clients)**	**100.0 (71)**	**100.0 (163)**	**100.0 (178)**	**100.0 (190)**	**100.0 (602)**	

Disclosed status to regular partner (among those with regular partner, *N*=497)	91.4 (53)	83.3 (125)	88.8 (127)	86.3 (126)	86.7 (431)	0.369
**Total (with regular partner)**	**191.4 (58)**	**183.3 (150)**	**188.8 (143)**	**186.3 (146)**	**186.7 (497)**	

**Figure 1:**
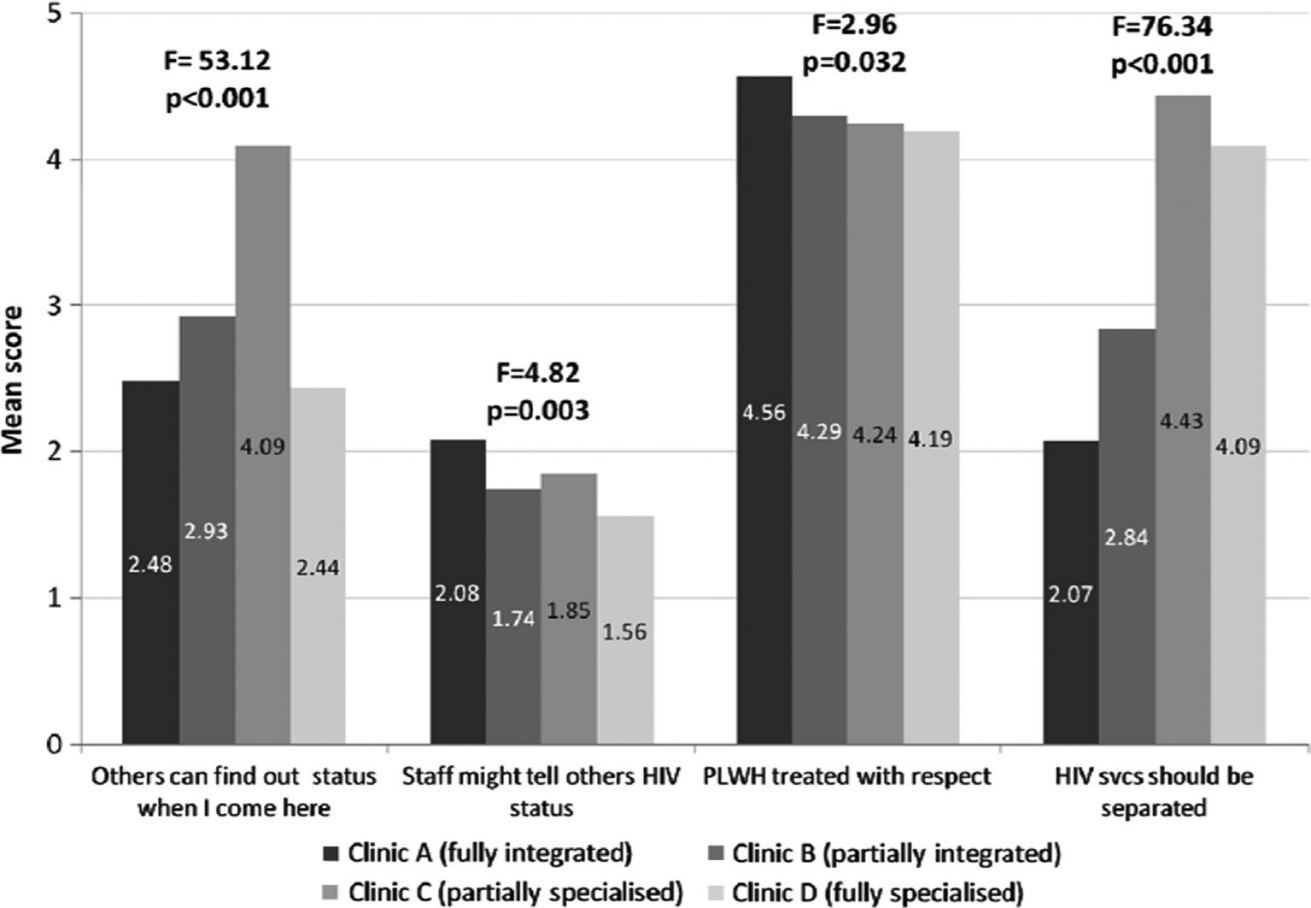
Stigmatization in the clinic (*N*=602).

Figure 1 presents ratings of stigmatizing experiences within the clinic. There was a general perception that HIV status could be exposed through clinic attendance (“Others can find out my status when I come here”) (mean 3.06/5.00, SD 1.5). Clients at Clinic C, the partially stand-alone hospital model, were particularly concerned (4.09, SD 1.3). However, clients at Clinic D felt most protected with their status, despite being in an HIV-only facility (2.44, SD 1.4). Most clients, across all sites, trusted staff to maintain their status confidentially, with low fears of gossiping (1.76, SD 1.1); and almost all felt strongly that staff treated PLWH with respect (4.28, SD 0.9). There were significant differences in client feelings on HIV service specialization (“It's better if HIV services are separated from other health services”) (*F*=76.3, *p*<0.001) with those at stand-alone sites feeling strongly that these services should be kept separate (4.43 and 4.09 at Clinics C and D, respectively).

In a crude analysis, factors with some evidence of association with fear of status exposure (*p*<0.15) included clinic model, sex, age, income and costs to reach clinic. Table 4 shows the results of the multivariable analysis on perceived HIV status exposure. The total proportion of clients with a high perceived exposure risk (≥4.00/5.00) was 44%. After controlling for all other variables in the table, the odds of perceived exposure were higher at the partially integrated site, Clinic B, compared to the fully stand-alone site, Clinic D (aOR 3.33, 95% CI 1.98–5.60), and were much higher at Clinic C, the partially stand-alone hospital model (aOR 11.84, 95% CI 6.89–20.36). There was no statistical difference in perceived exposure between clients at the most integrated and most stand-alone site (A and D). Other factors associated with fear of exposure at the clinic (p<0.05) were younger age; living further away from the clinic (in cost terms); time enrolled at clinic; and not being on TB treatment. Sex was not associated with fear of HIV status exposure (p>0.05).

**Table 4:** Crude and adjusted odds of fearing exposure of HIV status through clinic attendance (*N*=602)

		Perceived exposure score≥4					
						
Variable	Category	N	N (%)	cOR	95% CI	aOR*	95% CI
Clinic model	Clinic A	71	16 (22.5)	1.13	(0.58–2.18)	0.92	(0.45–1.89)
	Clinic B	163	74 (45.4)	3.22	(2.02–5.14)	3.33	(1.98–5.60)
	Clinic C	178	135 (75.8)	12.16	(7.43–19.87)	11.84	(6.89–20.36)
	Clinic D	190	39 (20.5)	1.00	—	1.00	—
Age group	Less than 25	99	42 (42.4)	1.00	—	1.00	—
	25–29	137	64 (46.7)	1.19	(0.71–2.00)	1.13	(0.62–2.06)
	30–39	225	107 (47.6)	1.23	(0.76–1.98)	1.13	(0.64–1.98)
	40 or over	141	51 (36.2)	0.77	(0.45–1.30)	0.55	(0.29–1.06)
Distance from clinic (cost)	E0–E5	274	114 (41.6)	1.00	—	1.00	—
	E6–E10	143	56 (39.2)	0.90	(0.60–1.37)	0.92	(0.57–1.49)
	E11–E20	122	58 (47.5)	1.27	(0.83–1.95)	1.13	(0.67–1.93)
	>E20	63	36 (57.1)	1.87	(1.08–3.26)	2.97	(1.53–5.78)
Time enrolled at clinic	<6 months	228	96 (42.1)	1.00	—	1.00	—
	6 months–2 years	264	93 (35.2)	0.75	(0.52–1.08)	0.89	(0.58–1.37)
	>2 years	110	75 (68.2)	2.95	(1.82–4.76)	2.04	(1.17–3.59)
On ARVs	No	107	38 (35.5)	0.66	(0.42–1.01)	0.67	(0.40–1.11)
	Yes	495	226 (45.7)	1.00	—	1.00	—
TB treatment	No treatment	574	254 (44.3)	1.00	—	1.00	—
	On treatment	28	10 (35.7)	0.70	(0.32–1.54)	0.40	(0.16–1.00)

* Adjusted for all other variables in table.

### Qualitative results

Table 5 summarizes the key background characteristics of the qualitative sample.

**Table 5:** Overview of qualitative client sample

Characteristic	Category	No. of respondents (*N*=22)
Clinic (round 2/3 in brackets)	Clinic A	5 (4)
	Clinic B	6 (5)
	Clinic C	5 (3)
	Clinic D	6 (4)
Sex	Male	7
	Female	15
Mean age (range)		31 (22–45)
Pregnancy status (f)	Pregnant	5
	Not pregnant	10
Education	Primary	7
	Secondary	12
	College or above	2
	Adult education	1
Monthly household income (SLZ)	<500	3
	500–1000	6
	1000–3000	9
	3000–5000	3
	>5000	1

The data suggested that most clients, across all models of care, felt (or had felt) discomfort about attending ART clinics, caused primarily by fear of status exposure at the clinic and concern about bumping into acquaintances. However, this anxiety was also related to clients’ own acceptance of their HIV status; those experiencing greater discomfort were those who had failed to disclose their status to partners, family or friends. Acceptance of status and increasing comfort in HIV clinics were seen as a gradual progression. The data also suggested that the relationship between clinic model, privacy and fear of status exposure was complex. For some, integrated care helped to protect their HIV status, in particular when it was perceived that there was no specific room for ART services:

someone who came for treatment sits on the same bench [as others] […] which is different to other hospitals where it's even written or labelled on the doors and then it's obvious that you're going for [HIV] services. So here […] it's only me and the doctor who know what I've come for. [Female, Clinic A]

Integrated care implied that you could “blend with the rest” and avoid identification as a PLWH. Clinic A, where there was no specific HIV nurse or doctor, was found to be particularly effective at delivering this benefit, and several clients there selectively chose that site due to concerns over privacy and confidentiality.

However, HIV status could still be revealed at integrated clinics. Indicative signs included collecting food parcels (only for ART clients), carrying green ART registration cards, attending the doctor's room or collecting ART drugs. Thus, discomfort was experienced, even at Clinic A:

It does get uncomfortable [waiting], especially when there are a lot of people in there. Sometimes even if people don't ask you, you feel like they somehow know why you came here. And then there's the food they give you right when you walk out. I've decided I'm not taking the food again …. [Female, Clinic A]

ART provision in specified rooms at Clinic B seemed particularly stigmatizing, implying that “people can see that that queue is for those who are positive.” While clinic staff had made an effort to label the ART room “Room 3,” to reduce stigma, providers could still exacerbate status exposure risk:

[They] announced that those who were there to get pills needed to go to Room 3 [… It] was really bad because everyone was just sitting in the waiting room, and nobody was paying attention to what others were there for … then all of a sudden we have to get up because we're the ones that've been called. People didn't need to know …. [Female, Clinic B]

Conversely, at stand-alone sites there seemed to be lower levels of discomfort. Many clients felt their status was protected there *because* everybody else was positive. Clients, who formerly hid their pills, felt free to be open:

At first I'd put the pills in a plastic bag so they couldn't see them, but now I don't care […] I feel free and at the hospital when you meet others like yourself you talk, and they're also not afraid anymore, we no longer discriminate against each other. [Male, Clinic C]

Many clients at these sites reported gaining encouragement from other PLWH; conversations about health status were therapeutic and also supported drug adherence. For some, such interactions were the only opportunity to be open about their HIV status, since disclosure at home was impossible. This support contrasted with experiences of those at integrated sites; for example, one client at Clinic B was keen to chat with other PLWH but found it too difficult to identify them.

Aspects of clinic reorganization (occurring either before or during the study) also influenced status exposure. At fully integrated Clinic A, clients could collect drugs *inside* the dispensary, rather than through a public window; new filing systems were developed so that all clients used the same coloured files; a new drugs form replaced the green ART patient cards; and a triaging nurse was hired who would see clients in private. At Clinic D, the ART waiting room had been separated from the voluntary counselling and testing of HIV (VCT) waiting room to increase confidentiality, and the clinic had been labelled a “Help Centre” rather than “HIV clinic.”

## Discussion

This study has demonstrated that stigma associated with HIV status exposure was influenced by the organization of care, though not always in ways that had been anticipated. While integrated sites increased confidentiality for some clients, this benefit was not universal. Aspects of service organization that breeched confidentiality included name-calling, room labelling, providing food packages, the use of ART patient cards and drug dispensing systems. After controlling for client-level differences between sites, those at partially integrated Clinic B had over three times greater odds of perceiving status exposure than those at the HIV-only clinic, Clinic D. Clients at the hospital model also strongly perceived exposure risk (over 13 times greater odds than Clinic D), most likely due to the location of the “ART building” nearby other hospital departments. Therefore, partially integrated/stand-alone models entailed the greatest risk of status exposure for HIV clients.

However, many clients at stand-alone sites were not bothered if others knew what they came in for, and most desired ART to remain separated from other health services (in contrast to clients at integrated sites). Comfort in stand-alone sites was partly attributable to mutual support gained from other PLWH in waiting rooms, an effect that has been found in a study of ART decentralization in Zambia [20]. However, this benefit was generally unavailable to those in integrated sites. It is conceivable that by “forcing” status exposure through clinic attendance, HIV-only services were contributing to a therapeutic process, that is, the psycho-social benefits of disclosure that have been noted elsewhere [8]. The strong preferences among clients at stand-alone sites to maintain dedicated HIV services is interesting and underscores similar findings from a study of South African ART clinics which demonstrated fears about decentralization of ART to primary care [39]. While not documented in this article, the broader study found that part of this preference also stems from perceptions of higher quality care in ART units compared to outpatient facilities [34], a finding also noted elsewhere [40]. Strategies to integrate HIV care with PHC and hospital out-patient services may therefore prove unpopular with HIV clients and furthermore have the potential to increase HIV-related stigmatization if not properly managed.

Several study limitations should be acknowledged. First, the observational design precludes determination of causality between exposure (to clinic model) and stigma outcomes. While longitudinal research is required to establish a more robust association, the additional use of qualitative methods here aimed to compensate for this weakness by exploring user perceptions on the way that the model of care impacted on felt stigma. The high degree of congruence across cases and repeated interviews underscore the reliability of these data and interpretations made. Second, the survey may have suffered from response bias: refusal rates were higher at Clinic A and Clinic B. While no further data were captured on this group, stigma could have been a reason for refusing interview, and thus stigma measurements at these sites may be underestimated. Third, the study suffers from selection bias in the absence of client randomization across clinics. While the use of a multivariable model attempted to control for confounding in the quantitative sample, it is unlikely to have completely eliminated it. Finally, the small number of clinics studied from one town in Swaziland restricts generalizability of the findings. However, the case study approach taken with this study aimed to provide an in-depth understanding of health care experiences and processes, allowing lessons to be learned for other similar settings with a high HIV burden.

It is also important to note that this study has only reviewed one potential outcome of service integration. The broader study in which the research was undertaken has investigated other potential benefits, including access to SRH services and client satisfaction [34], while the larger Integra Initiative also evaluates integration processes outcomes, including provider perceptions on service integration, resource-use efficiency and health outcomes, across multiple clinics and countries. Any evaluation of specific models of care needs to consider the totality of potential benefits and risks of service integration.

## Conclusions

To conclude, the organization of care influences felt HIV stigma within clinics but does not determine it. Policy-makers and programme managers can take steps to reduce HIV-related stigma caused by structural factors, including within stand-alone HIV services. Successful strategies include careful clinic and room labelling, ensuring that HIV client records are unidentifiable, dispensing drugs either in private or without easy identification, finding discreet ways to deliver food packages to PLWH and separating waiting areas of VCT and ART clients at HIV-only clinics. ART clinics also have the potential to further diminish stigma by allowing PLWH to share experiences and coping mechanisms with others facing similar health and social problems and to benefit from the therapeutic process of disclosure. Such mechanisms need to be promoted at integrated sites where clients may not recognize other clients living with HIV.

Ongoing debates about the integration of HIV care within PHC services should therefore take cognizance of the potential benefits that stand-alone models of care may offer, in particular in high HIV prevalence settings where treatment needs have the potential to overwhelm the PHC system.

## References

[1] Criel B, De Brouwere V, Dugas S (1997). Integration of vertical programmes in multi-function health services.

[2] Osborne CM, van Praag E, Jackson H (1997). Models of care for patients with HIV/AIDS. AIDS.

[3] Matovu JK, Makumbi FE (2007). Expanding access to voluntary HIV counselling and testing in sub-Saharan Africa: alternative approaches for improving uptake, 2001–2007. Trop Med Int Health.

[4] Bradley H, Bedada A, Tsui A, Brahmbhatt H, Gillespie D, Kidanu A (2008). HIV and family planning service integration and voluntary HIV counselling and testing client composition in Ethiopia. AIDS Care.

[5] Tollman SM, Kahn K, Sartorius B, Collinson MA, Clark SJ, Garenne ML (2008). Implications of mortality transition for primary health care in rural South Africa: a population-based surveillance study. Lancet.

[6] Nyblade L, Stangl A, Weiss E, Ashburn K (2009). Combating HIV stigma in health care settings: what works?. J Int AIDS Soc.

[7] UNAIDS (2003). UNAIDS fact sheet on stigma and discrimination.

[8] Greeff M, Phetlhu R, Makoae LN, Dlamini PS, Holzemer WL, Naidoo JR (2008). Disclosure of HIV status: experiences and perceptions of persons living with HIV/AIDS and nurses involved in their care in Africa. Qual Health Res.

[9] Stringer EM, Sinkala M, Stringer JS, Mzyece E, Makuka I, Goldenberg RL (2003). Prevention of mother-to-child transmission of HIV in Africa: successes and challenges in scaling-up a nevirapine-based program in Lusaka, Zambia. AIDS.

[10] Weiser S, Wolfe W, Bangsberg D, Thior I, Gilbert P, Makhema J (2003). Barriers to antiretroviral adherence for patients living with HIV infection and AIDS in Botswana. J Acquir Immune Defic Syndr.

[11] Thorsen VC, Sundby J, Martinson F (2008). Potential initiators of HIV-related stigmatization: ethical and programmatic challenges for PMTCT programs. Dev World Bioeth.

[12] Otieno PA, Kohler PK, Bosire RK, Brown ER, Macharia SW, John-Stewart GC (2010). Determinants of failure to access care in mothers referred to HIV treatment programs in Nairobi, Kenya. AIDS Care.

[13] Mahajan AP, Sayles JN, Patel VA, Remien RH, Sawires SR, Ortiz DJ (2008). Stigma in the HIV/AIDS epidemic: a review of the literature and recommendations for the way forward. AIDS.

[14] Mill JE, Edwards N, Jackson RC, MacLean L, Chaw-Kant J (2010). Stigmatization as a social control mechanism for persons living with HIV and AIDS. Qual Health Res.

[15] Best K (2004). Cambodia: clients find everything they need in one place. Network.

[16] IPPF (2005). Models of care project: linking HIV/AIDS treatment, care and support in sexual and reproductive health care settings, examples in action.

[17] Maharaj P, Cleland J (2005). Integration of sexual and reproductive health services in KwaZulu-Natal, South Africa. Health Policy Plan.

[18] IPPF (2006). La integración de los servicios de atención y tratamiento del VIH/SIDA en los ámbitos de salud reproductiva.

[19] Orner P, Cooper D, Myer L, Zweigenthal V, Bekker LG, Moodley J (2008). Clients’ perspectives on HIV/AIDS care and treatment and reproductive health services in South Africa. AIDS Care.

[20] Topp SM, Chipukuma JM, Giganti M, Mwango LK, Chiko LM, Tambatamba-Chapula B (2010). Strengthening health systems at facility-level: feasibility of integrating antiretroviral therapy into primary health care services in lusaka, Zambia. PLoS One.

[21] Adamchak SE, Grey TE, Otterness C, Katz K, Janowitz B (2007). Introducing family planning services into antiretroviral programs in Ghana: an evaluation of a pilot intervention.

[22] Nyamuryekung'e K, Laukamm-Josten U, Vuylsteke B, Mbuya C, Hamelmann C, Outwater A (1997). STD services for women at truck stop in Tanzania: evaluation of acceptable approaches. East Afr Med J.

[23] EngenderHealth, UNFPA (2006). Sexual and reproductive health needs of women and adolescent girls living with HIV: research report on qualitative findings from Brazil, Ethiopia and the Ukraine, July 2006.

[24] PATH (2007). Options and challenges for converging HIV and sexual and reproductive health services in India: findings from an assessment in Andhra Pradesh, Bihar, Maharashtra, and Uttar Pradesh.

[25] Mayhew SH (2000). Integration of STI services into FP/MCH services: health service and social contexts in rural Ghana. Reprod Health Matters.

[26] Ndhlovu L, Searle C, Miller R, Fisher A, Snyman E, Sloan N (2003). Reproductive health services in KwaZulu Natal, South Africa: a situation analysis study focusing on HIV/AIDS services.

[27] CSO (2008). Swaziland Demographic and Health Survey 2006–07.

[28] PHR (2007). Epidemic of inequality: women's rights and HIV/AIDS in Botswana and Swaziland. an evidence-based report on the effects of gender inequity, stigma and discrimination.

[29] Shamos S, Hartwig KA, Zindela N (2009). Men's and women's experiences With HIV and stigma in Swaziland. Qual Health Res.

[30] Root R (2010). Situating experiences of HIV-related stigma in Swaziland. Glob Public Health.

[31] SWANNEPHA, FLAS (2011). Assessment of the stigma index among people living with HIV and AIDS in Swaziland.

[32] Kamiru H, Bruce K, Okello V, Mndzebele S, Vandelanotte J, Preko P (2010). Increasing access to ART care and treatment through decentralisation: early lessons from the Swaziland National AIDS Program and ICAP experience.

[33] NERCHA (2009). National strategic framework for HIV and AIDS 2009–2014.

[34] Church K (2011). Meeting the sexual and reproductive health needs of HIV care and treatment clients in Swaziland: a comparative case study of integrated and stand-alone models of care [PhD Thesis].

[35] Mayhew SH, Vassall A, Kimani JK, Church K, Obure CD, Friend du-Preez N (2012). Study protocol for the integra initiative to assess the benefits and costs of integrating sexual and reproductive health and HIV services in Kenya and Swaziland. BMC Public Health.

[36] Yin R (2003). Case study research, Third Edition.

[37] Gilson L Considering context in health systems research.

[38] Glaser BG, Strauss AL (1999). Discovery of grounded theory: strategies for qualitative research.

[39] Mukora R, Charalambous S, Dahab M, Hamilton R, Karstaedt A (2011). A study of patient attitudes towards decentralisation of HIV care in an urban clinic in South Africa. BMC Health Serv Res.

[40] Nabbuye-Sekandi J, Makumbi FE, Kasangaki A, Kizza IB, Tugumisirize J, Nshimye E (2011). Patient satisfaction with services in outpatient clinics at Mulago hospital, Uganda. Int J Qual Health Care.

